# Effectiveness and safety of hydrogen inhalation therapy as an additional treatment for hypertension in real-world practice: a retrospective, observational study in China

**DOI:** 10.3389/fcvm.2024.1391282

**Published:** 2024-11-12

**Authors:** Hongxiang Ji, Hualin Sun, Yinghui Zhang, Ziyi Zhao, Xin Gao, Chunhe Wang, Yang Yang, Xiaodong Zhang, Jianyong Gao, Dequan Man, Qian Yang, Ying Yang, Chengbin Yue, Changjiang Chen, Xiaoheng Ding, Tongshang Ni

**Affiliations:** ^1^ The First Clinical Medical College, Shandong University of Traditional Chinese Medicine, Jinan, Shandong, China; ^2^ Graduate School, Chengde Medical University, Chengde, Hebei, China; ^3^ Nursing Department, Qingzhou People's Hospital, Qingzhou, Shandong, China; ^4^ Department of Hand and Foot, Microsurgery, The Affiliated Hospital of Qingdao University, Qingdao, Shandong, China; ^5^ School of Health Management, Hengxing University, Qingdao, Shandong, China; ^6^ Center of Integrated Traditional Chinese and Western Medicine, School of Basic Medicine, Qingdao University, Qingdao, Shandong, China

**Keywords:** hypertension, observational study, hydrogen inhalation, blood pressure, real-world study

## Abstract

**Aim:**

To evaluate the real-life effectiveness and safety of hydrogen inhalation (HI) therapy as an additional treatment in Chinese adults with hypertension.

**Methods:**

This observational, retrospective clinical study included hypertensive patients receiving routine antihypertensives with or without HI initiation from 2018 to 2023. Participants were assigned to the HI group or non-HI group (control group) after propensity score matching. The changes in mean systolic blood pressure (SBP) level during the 24-week follow-up period in different groups were examined primarily. The secondary outcome was the changes in diastolic blood pressure (DBP) and blood pressure (BP) control rate during the study. Several subgroup and sensitivity analyses were performed to confirm the robustness of our main findings. Adverse event (AE) was also assessed in patients of both groups.

**Results:**

In total, we selected 2,364 patients into the analysis. Both mean SBP and DBP levels significantly decreased in the HI group compared to control group at each follow-up visit with the between group difference of −4.63 mm Hg (95% CI, −6.51 to −2.74) at week 8, −6.69 mm Hg (95% CI, −8.54 to −4.85) at week 16, −7.81 mm Hg (95% CI, −9.57 to −6.04) at week 24 for SBP, and −1.83 mm Hg (95% CI, −3.21 to −0.45) at week 8, −2.57 mm Hg (95% CI, −3.97 to −1.17) at week 16, −2.89 mm Hg (95% CI, −4.24 to −1.54) at week 24 for DBP. Patients in the HI group were more likely to attain controlled BP at the follow-up period with odds ratio of 1.44 (95% CI, 1.21–1.72) at week 8, 1.90 (95% CI, 1.59–2.27) at week 16, and 2.24 (95% CI, 1.87–2.68) at the end. The trends of subgroup and sensitivity analyses were mostly consistent with the main analysis. The incidences of AEs were similar between the HI group and control group with all *p*-value >0.05.

**Conclusion:**

The HI therapy is related to significant amelioration in BP levels with acceptable safety profile in Chinese hypertensive adults after 24 weeks of treatment, building a clinical ground for further research to evaluate the antihypertensive effect of HI therapy.

## Introduction

It is estimated that the number of hypertensive patients worldwide will reach 1.56 billion by 2025, and the Chinese morbidity was 23.2%–44.7% in 2015 (1, 2). However, under the current medical care, the hypertension control rates are usually low in populations of low- and middle-income countries, with merely 10% patients having the capacity of gaining controlled blood pressure (BP) (3, 4). Based on a study of 1.7 million adults between 2014 and 2017, hypertensive patients achieving control merely accounted for 7.2% in China (5). Therefore, further developing effective therapy for hypertension is rather essential.

Molecular hydrogen, as an emerging medical gas with comprehensive effects of rapid diffuse, anti-apoptotic, anti-inflammatory, and antioxidant, has shown favorable effects to various diseases (6, 7). Numerous studies have reported that the application of hydrogen is therapeutic for neurodegeneration (8, 9), cerebral infarction (10, 11), chronic obstructive pulmonary disease (12, 13), metabolic syndrome (14, 15), non-alcoholic fatty liver disease (16, 17), cancer (18, 19). Furthermore, hydrogen has demonstrated beneficial effect on cardiovascular system. In animal models, it was reported that 4-week hydrogen inhalation (HI) led to therapeutic effect for hypertensive rats (20). The left ventricular hypertrophy was alleviated by hydrogen-rich saline injection or HI in rats with various hypertension (21, 22). The renal injury was also improved by hydrogen-rich water (HRW) intake in hypertensive rats (23). In clinical studies, the BP control was ameliorated by hydrogen-rich dialysate in patients with chronic dialysis (24). Recently, a randomized, placebo-controlled trial has revealed that maintaining HI therapy 4 h per day for 2 weeks demonstrates marked BP lowering effect in 60 Chinese hypertensive patients (25).

The hydrogen medicine is receiving extensive attention globally, especially in east Asia countries such as China and Japan. Ever-growing number of Chinese patients with metabolic disorders has acknowledged the therapeutic effects of molecular hydrogen. There are substantial Chinese hypertensive patients applying HI therapy in order to improve their BP levels.

Despite randomized controlled trial (RCT) is widely recognized as the golden standard, the relatively small sample size and short follow-up period of previous studies greatly limit their findings to examine the antihypertensive effect of HI treatment. Therefore, we performed a retrospective study to assess whether the 24-week HI therapy is effective and safe as an additional treatment for Chinese patients with hypertension in real life clinical practice.

## Method and materials

### Study design and patient population

Herein, a multicenter, retrospective, double-arm study evaluated the effectiveness and safety of HI in Chinese hypertensive patients in real-life setting. This study was performed on the basis of a medical records database which had been previously described in detail (26). In short, the health records database documents and stores clinical data via electronic medical record, which contains basic demographics, vitals, laboratory values, diagnostic reports, pharmacy dispenses, etc., from several hospitals and health examination centers in Qingdao, China. Study numbers were utilized to de-identify eligible participants.

Hypertensive patients who initiated HI therapy or received routine hypotensive treatment for at least 6 months from January 1, 2018 to January 1, 2023 were eligible for this study. Subjects were divided into two groups: the HI group or non-HI group (control group), as per former treatment details. All subjects were treated with routine therapeutic standard according to their attending physicians’ discretion, while patients in the HI group additionally initiated and continuously maintained HI therapy. The initiation of HI therapy was defined as the absence of molecular hydrogen intervention by any means in the previous 52 weeks. Participants’ medical data extracted from electronic medical records and their HI conditions during the study were connected by individualized identification.

The inclusion criteria were: (1) ≥18 years old. (2) BP ≥140/90 mm Hg or receiving antihypertensive treatment simultaneously. (3) Without change in recorded antihypertensive medication during the 6-month follow-up period. (4) ≥1 clinical measurement at baseline period and one of the visit points at follow-up period. (5) Maintaining HI duration ≥15 h per week for subjects in HI group. Patients were excluded if they were: (1) secondary hypertension. (2) Pregnant or breastfeeding female. (3) Serious disorders with a poor prognosis, including advanced renal dysfunction or severe liver dysfunction. (4) Entering hospice at the follow-up period. (5) Concurrently participation in any investigational research demanding interventions apart from routine therapeutic practice.

### Hydrogen inhalation protocol

Since the therapeutic effects of hydrogen therapy are widely recognized by Chinese public, hypertensive patients received HI therapy voluntarily in the specific hydrogen treatment departments. Experienced health professionals of hydrogen medicine provided specialized knowledge (the benefit and mechanism of hydrogen molecule, detailed operation procedures and points for attention of HI, etc.) to each participant, ensuring the valid implementation of hydrogen therapy. Individuals were treated with hydrogen gas therapy at their convenience without specific time point. Health technicians were assigned to monitor the entire process of HI and record personally identifiable information (name, resident identity card number, medical insurance card number, etc.) together with hydrogen gas intake conditions (each duration of HI, flow rate, etc.) for all patients. All health workers and researchers were well trained and tested systematically each year to remain the professionalism. No other intervention (diet suggestion, exercise management, etc.) was provided for individuals receiving HI.

Subjects utilized nasal tube to inhale pure hydrogen gas at the flow rate of 2.0 L/min, which was provided by hydrogen-producing machine (Qingdao Haizhisheng Corp., Ltd., Qingdao, China). Although specialists in hydrogen medicine would make individualized recommendation of HI duration for each patient, the time length of each inhalation and the frequency were based on individual decision, in view of the high safety profile of hydrogen gas (27). The flow rate of 2.0 L/min was chosen as it was most frequently applied by hypertensive subjects, as well as other studies (13, 16, 18, 28–31).

### Data collection and assessment

The demographic and clinical data were taken at 4 visit points in 24-week follow-up period: V1, i.e., baseline (prior to index date), V2 (8 ± 2 weeks post index date), V3 (16 ± 2 weeks post index date), V4 (24 ± 2 weeks post index date). The timepoint of HI therapy initiation was defined as index date.

Baseline characteristics including sex, age, body mass index (BMI), heart rate, smoking and drinking status, family history of disease, creatinine, estimated glomerular filtration rate (eGFR), medical comorbidity, and concomitant medications were analyzed at baseline. The concomitant antihypertensives, BP measurements, and application status of HI were assessed at each visit point. BMI was calculated via dividing weight (kg) by height squared (m^2^). The eGFR was determined by the creatinine equation of Chronic Kidney Disease Epidemiology Collaboration (32). BP was taken by electronic sphygmomanometer in seated patients after ≥5 min of rest. To maintain accuracy, BP response underwent manual inspection if the value was ≥2 standard deviation (SD) from the mean. The average value was applied if BP was measured more than once on the same date, and clinical data most proximal to baseline or each visit point were evaluated.

The incidence of adverse event (AE) including no. of events, no. of patients, and incidence rate during 24-week follow-up period was screened and selected from physicians’ recordings to evaluate the safety outcome of HI.

The primary endpoint of this study was the changes in systolic blood pressure (SBP) levels from baseline to follow-up visits. The secondary endpoint was the changes in diastolic blood pressure (DBP) and BP control rate up to 24 weeks after the index date. The BP control rate was defined as the proportion of patients maintaining BP <140/90 mm Hg.

### Ethics approval

The Ethics Committee of Hengxing University approved this study. Written informed consents were collected from all the patients. The study was executed in line with the revised Declaration of Helsinki.

### Statistical analysis

Due to the absence of prospective randomization, massive differences of baseline characteristics appeared in subjects between the HI group and control group. Thus, the propensity score matching (PSM) was performed to minimize selective bias and balance the cohorts. We established a logistic regression model according to all baseline variables in Table 1, evaluating the propensity scores of each treatment cohort. PSM was applied with a nearest neighbor 1:1 matched approach (without replacement) and a caliper of 0.10 SD (33).

**Table 1:** Baseline characteristics of patients after propensity score matching.

Parameters	HI group (*N* = 1,182)	Control group (*N* = 1,182)	*P*-value
Sex (female)	614 (51.9)	583 (49.3)	0.202
Age, years	63.71 (11.86)	63.27 (11.40)	0.363
BMI, kg/m^2^	25.47 (3.91)	25.43 (3.88)	0.837
SBP, mm Hg	144.49 (16.49)	144.39 (17.16)	0.888
DBP, mm Hg	90.42 (12.17)	89.58 (12.53)	0.099
Controlled BP^a^	236 (20.0)	244 (20.6)	0.683
Heart rate, bpm	74.30 (9.02)	73.96 (8.88)	0.363
Current smoker	296 (25.0)	292 (24.7)	0.849
Current drinker	274 (23.2)	269 (22.8)	0.807
Family history of hypertension	205 (17.3)	216 (18.3)	0.554
Serum creatinine, mg/dL	1.10 (0.30)	1.08 (0.31)	0.087
eGFR, mL/min/1.73 m^2^	84.86 (20.09)	85.32 (20.41)	0.580
Concomitant medications			
ACEIs/ARBs	777 (65.7)	747 (63.2)	0.197
Calcium channel blockers	553 (46.8)	574 (48.6)	0.387
Diuretics	150 (12.7)	139 (11.8)	0.490
Beta-blockers	282 (23.9)	298 (25.2)	0.444
Alpha-blockers	3 (0.3)	4 (0.3)	1.000
Antiplatelets	175 (14.8)	178 (15.1)	0.863
Statins	301 (25.5)	291 (24.6)	0.635
Antidiabetics	279 (23.6)	290 (24.5)	0.597
Anticoagulants	40 (3.4)	46 (3.9)	0.510
NSAIDs	93 (7.9)	95 (8.0)	0.879
Comorbidities			
Diabetes	361 (30.5)	354 (29.9)	0.754
Stroke or TIA	55 (4.7)	62 (5.2)	0.507
Chronic kidney disease	303 (25.6)	295 (25.0)	0.705
Dyslipidemia	421 (35.6)	413 (34.9)	0.731
Coronary heart disease	182 (15.4)	175 (14.8)	0.688
Heart failure	42 (3.6)	48 (4.1)	0.519
Atrial fibrillation	39 (3.3)	30 (2.5)	0.271
Peripheral vascular disease	10 (0.8)	11 (0.9)	0.826
Cancer	30 (2.5)	35 (3.0)	0.529
Depression	59 (5.0)	59 (5.0)	1.000

Note: All data are presented as mean (SD) or as *n* (%).

HI, hydrogen inhalation; BMI, body mass index; SBP, systolic blood pressure; DBP, diastolic blood pressure; BP, blood pressure; eGFR, estimated glomerular filtration rate; ACEIs angiotensin-converting enzyme inhibitors; ARBs angiotensin receptor blockers; NSAIDs, non-steroidal anti-inflammatory drugs; TIA, transient ischemic attack.

^a^ Controlled BP was considered as SBP <140 mm Hg and DBP <90 mm Hg.

Continuous data and categorical data were expressed as mean (SD) and frequency (percentage) separately. The changes of clinical values from baseline to different visit point in same group were assessed by paired *t*-tests. Meanwhile, the differences in changes of clinical parameters between different cohorts at same time point were evaluated by independent *t*-tests. Odds ratio (OR) with 95% CI was calculated to compare the proportion of patients maintaining controlled BP between two groups. χ^2^ test was performed to analyze the safety outcome of HI therapy. We performed these analyses on the available-case basis, excluding missing data.

Subgroup analysis was conducted based on several baseline characteristics including age (stratified by <65 and ≥65), sex (male vs. female), BMI (stratified by <25 kg/m^2^ and ≥25 kg/m^2^), diabetes mellitus (yes vs. no), SBP (stratified by <140 mm Hg and ≥140 mm Hg), antihypertension medication usage (≤1 vs. >1).

A series of sensitivity analysis were adopted to confirm the robustness of study findings: First, we settled the missing data at each visit point during the follow-up period by using a multiple-imputation method (34) (*N* = 305 in HI group and 287 in control group). Second, analyses were re-applied in all patients without performance of PSM (*N* = 1,734 in HI group and 2,392 in control group). Third, we performed a patient selection criterion with less restriction: the inclusion and exclusion criteria remained unchanged with the exception that participants with recording of change in antihypertensives during the study period were allowed (*N* = 1,773 in HI group and control group after PSM).

It required a sample size of 1,005 participants in each group to demonstrate statistically significant between-cohort difference of 2 mm Hg in SBP reduction with SD of 16.0 mm Hg, power of 80%, and in 0.05 two-sided significant level.

We used SAS 9.2 (SAS Institute) to conduct all analyses, and considered 2-sided *P* < 0.05 as statistically significant.

## Result

### General patients’ characteristics

**Figure 1:**
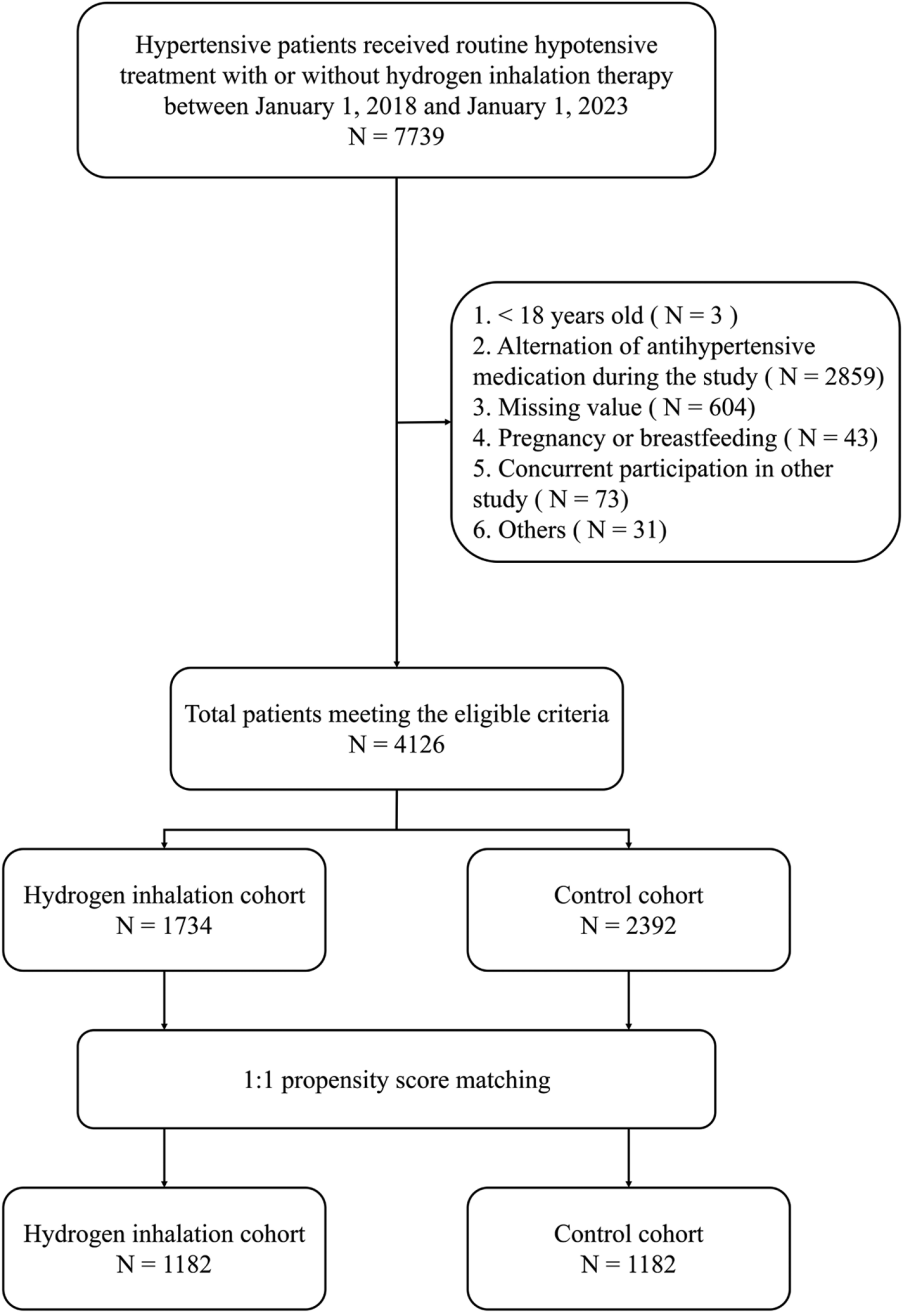
Flow chart of participant selection.

Based on the inclusion and exclusion criteria, a total of 4,126 patients were considered eligible for this study. 2,364 participants were selected for final analysis after PSM with 1,182 in HI group and 1,182 in control group (Figure 1). As shown in Table 1, patients’ baseline characteristics were similar between two groups after matching with mean age of 63.71 (11.86) vs. 63.27 (11.40) years and 51.9% vs. 49.3% female, separately. The mean BP was well balanced between HI group and control group with SBP: 144.49 (16.49) vs. 144.39 (17.16) mm Hg; DBP: 90.42 (12.17) vs. 89.58 (12.53) mm Hg; BP control rate: 20.0% vs. 20.6%. Other characteristics, such as BMI, heart rate, serum creatinine, eGFR, concomitant medications, and comorbidities were also comparable between two groups with all *p*-value >0.05.

Despite the antihypertensives remained invariant for all participants during the study, the alternations of dose were recorded (14.9% in HI group and 21.3% in control group) in 24-week follow-up period.

**Figure 2:**
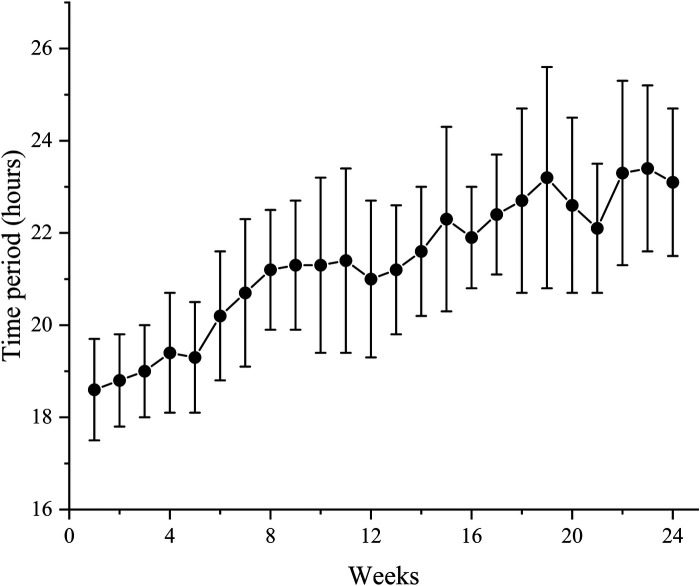
Mean time period of HI per week for all patients in HI group during the study. Data are shown as mean ± SD. HI, hydrogen inhalation.

In HI group, the mean time period of HI per week was 21.3 (2.2) h during the study. Patients inhaled hydrogen gas for 18.6 (1.1) h on average at week 1 and it reached 23.1 (1.6) h at week 24. Time period of HI per week demonstrated an increasing trend throughout the study (Figure 2).

### Effectiveness outcome

**Figure 3:**
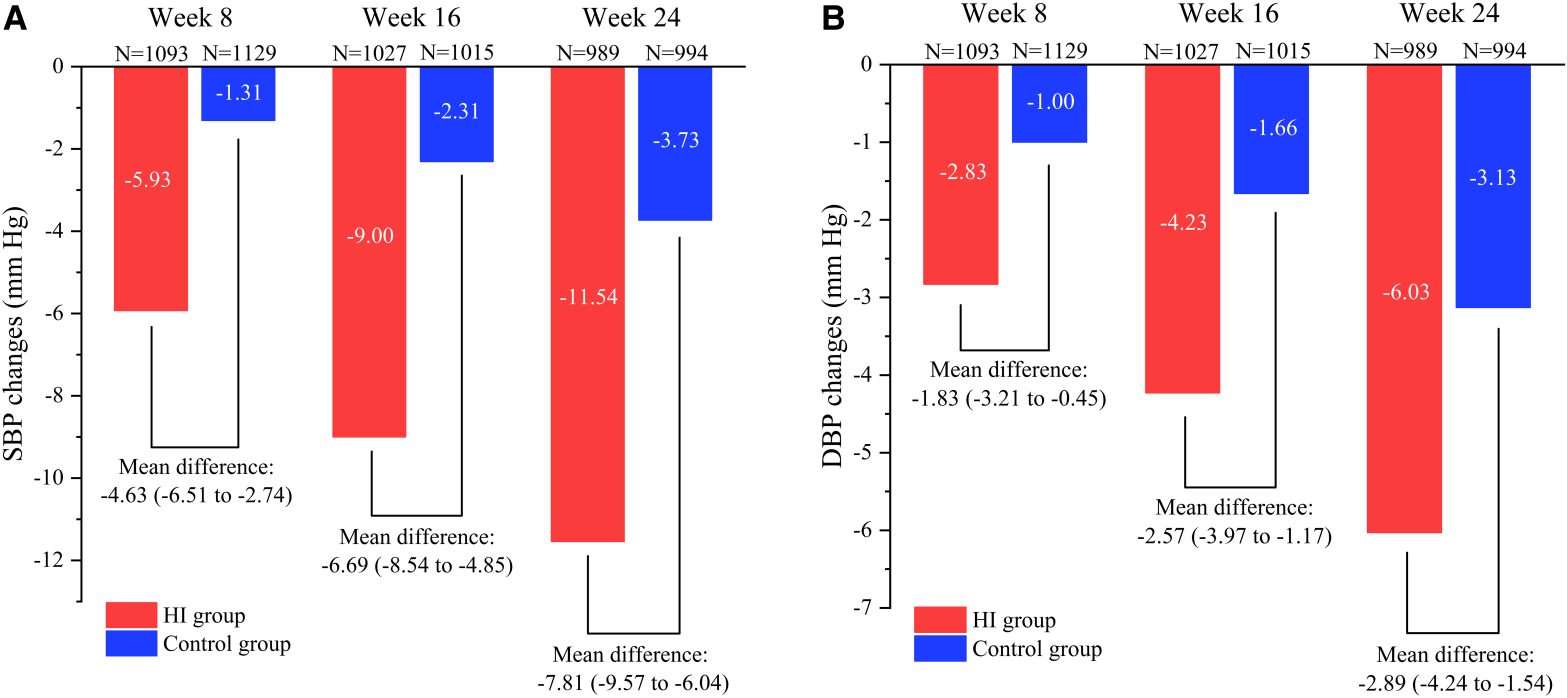
Comparison of reduction in SBP **(A)** and DBP **(B)** between HI group and control group from baseline to different visit point during the follow-up period. Data are shown as mean (95% CI). SBP, systolic blood pressure; DBP, diastolic blood pressure; HI, hydrogen inhalation.

**Figure 4:**
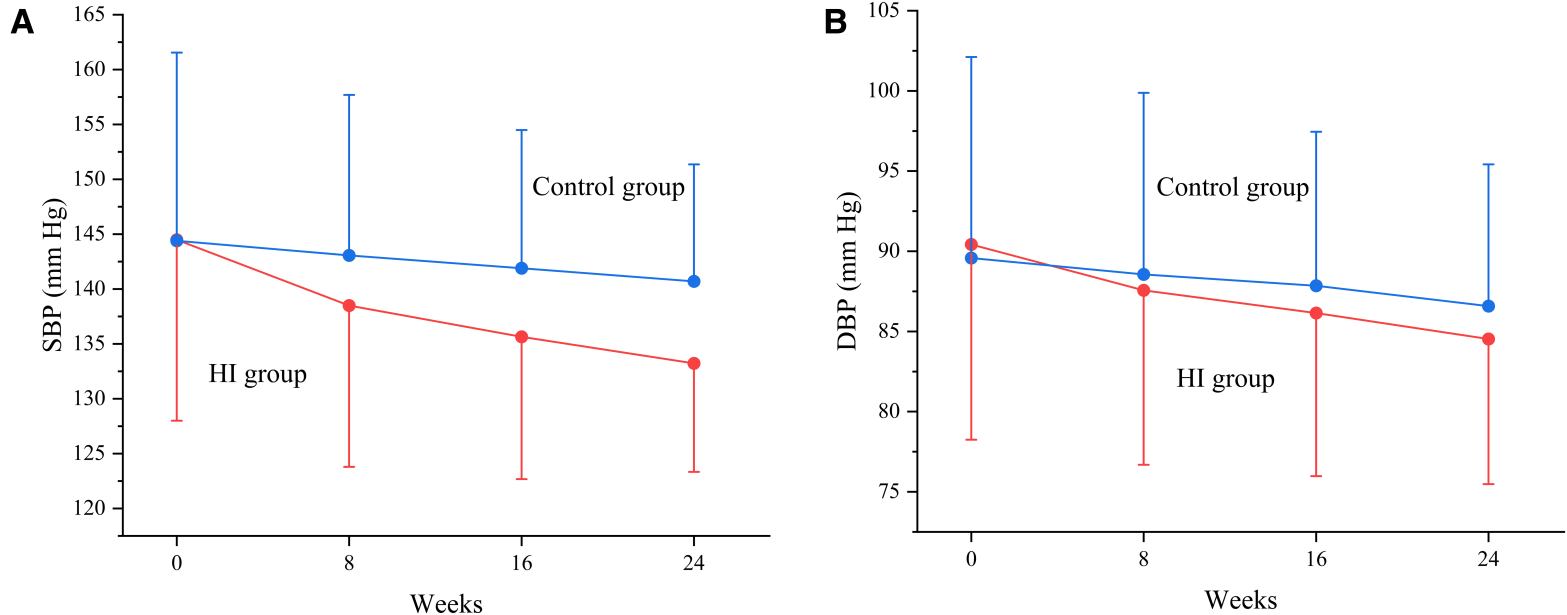
Change in SBP **(A)** and DBP **(B)** between HI and control groups from baseline to each visit point. Data are shown as mean ± SD. SBP, systolic blood pressure; DBP, diastolic blood pressure; HI, hydrogen inhalation.

The SBP levels decreased in HI group (−5.93 mm Hg; 95% CI, −7.30 to −4.57) and control group (−1.31 mm Hg; 95% CI, −2.61 to −0.00) at week 8 with the mean between-group difference of −4.63 mm Hg (95% CI, −6.51 to −2.74). At week 16, the mean SBP had reduced by −9.00 mm Hg (95% CI, −10.30 to −7.71) in HI group and −2.31 mm Hg (95% CI, −3.63 to −0.99) in control group. Meanwhile, the HI therapy demonstrated significantly favorable effect with between group difference of −6.69 mm Hg (95% CI, −8.54 to −4.85). Both the HI therapy and routine hypotensive treatment led to reduction in SBP (−11.54 mm Hg; 95% CI, −12.76 to −10.31 and −3.73 mm Hg; 95% CI, −5.00 to −2.46 respectively) by the end of week 24 and greater reduction was observed in HI group (−7.81 mm Hg; 95% CI, −9.57 to −6.04). The between-group difference in SBP increased during the study (Figures 3A, 4A).

At week 8, it demonstrated reductions of mean DBP level both in participants treated with HI therapy (−2.83 mm Hg; 95% CI, −3.83 to −1.84) and routine antihypertensives (−1.00 mm Hg; 95% CI, −1.97 to −0.04) with between-group difference of −1.83 mm Hg (95% CI, −3.21 to −0.45). The DBP had reduced by −4.23 mm Hg (95% CI, −5.21 to −3.24) in HI group and −1.66 mm Hg (95% CI, −2.65 to −0.66) in control group at week 16, while the HI therapy showed greater effect compared to control group (−2.57 mm Hg; 95% CI, −3.97 to −1.17). At week 24, the DBP levels decreased in HI group (−6.03 mm Hg; 95% CI, −6.98 to −5.08) and control group (−3.13 mm Hg; 95% CI, −4.09 to −2.17) with the mean between-group difference of −2.89 mm Hg (95% CI, −4.24 to −1.54). The between-group difference in DBP had increased as well during the study (Figures 3B, 4B).

**Figure 5:**
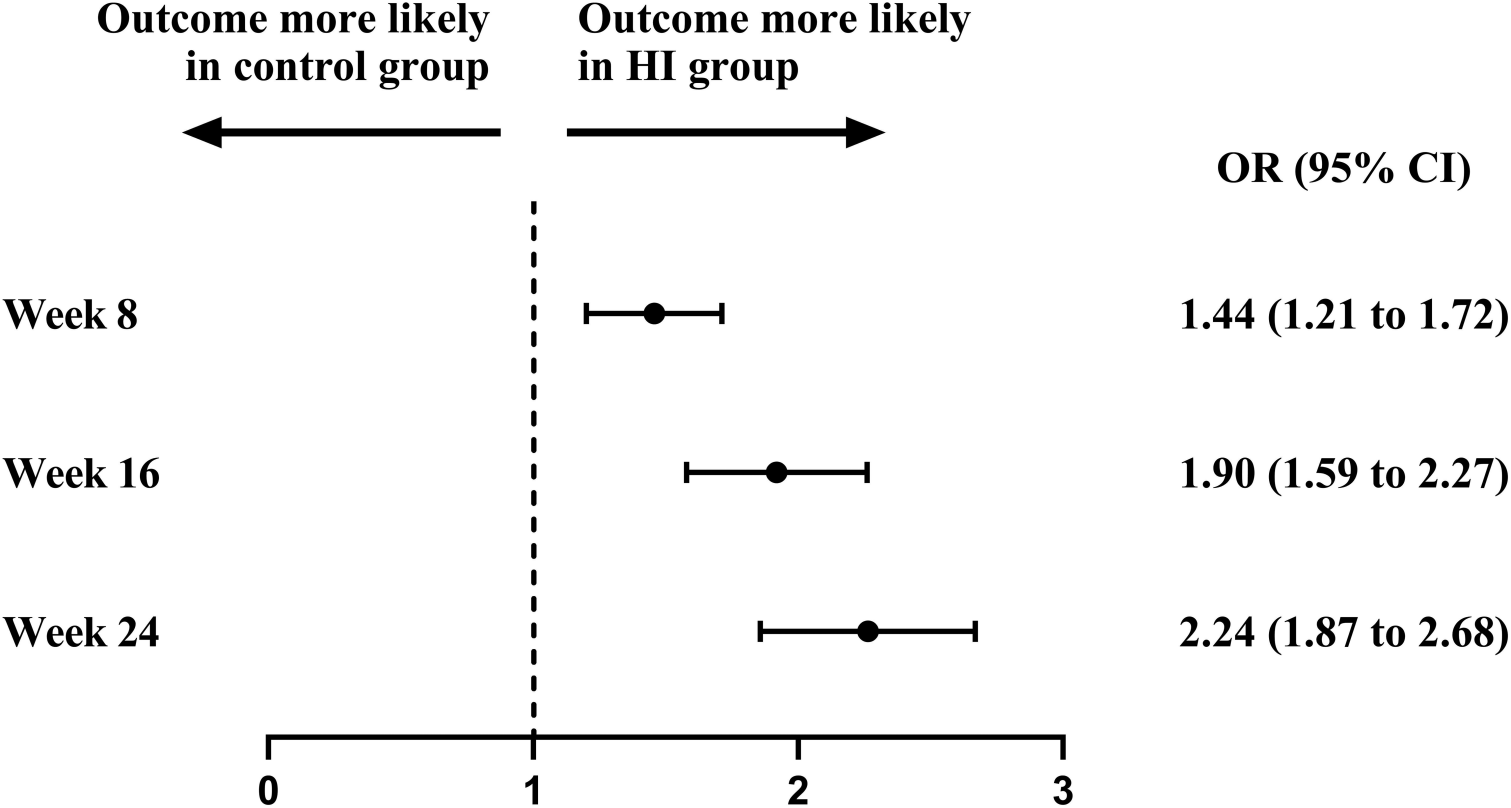
Attainment of controlled blood pressure between HI group and control group during follow-up period. Controlled blood pressure was defined as the blood pressure <140/90 mm Hg. HI, hydrogen inhalation; OR, odds ratio.

**Figure 6:**
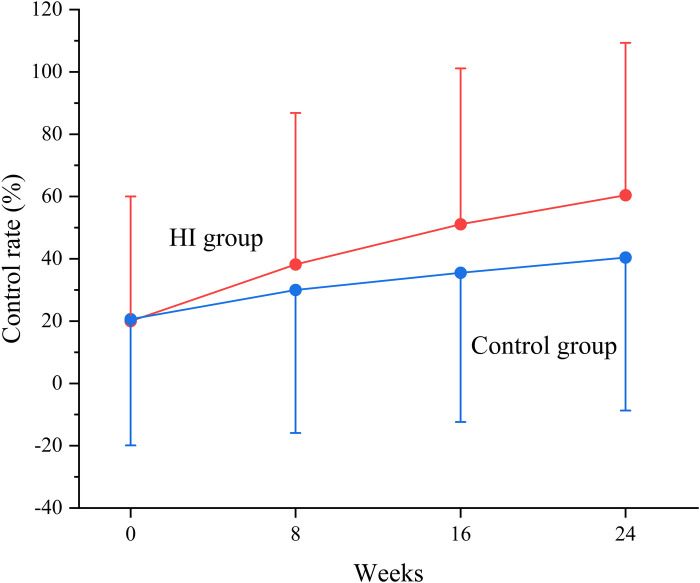
Change in blood pressure control rate between HI and control groups from baseline to each visit point. Data are shown as mean ± SD and controlled blood pressure was defined as the blood pressure <140/90 mm Hg. HI, hydrogen inhalation.

At baseline, the BP control rate was 20.0% in HI group and 20.6% in control group. The BP control rate reached 38.2% (95% CI, 35.4–41.1) at week 8, 51.1% (95% CI, 48.1–54.2) at week 16, and 60.4% (95% CI, 57.3–63.4) at week 24 in HI group. In control group, it increased to 30.0% (95% CI, 27.3–32.7) at week 8, 35.5% (95% CI, 32.5–38.4) at week 16, and 40.4% (95% CI, 37.4–43.5) at week 24. The likelihoods of attaining controlled BP were higher in HI group with OR of 1.44 (95% CI, 1.21–1.72) at week 8, 1.90 (95% CI, 1.59–2.27) at week 16, and 2.24 (95% CI, 1.87–2.68) by the end of week 24. The BP control rate demonstrated an increasing trend throughout the study for both groups (Figures 5, 6).

### Subgroup and sensitivity analysis

The results of subgroup and sensitivity analysis were consistent with the overall findings. Similar trends were found in prespecified subgroups including age, sex, BMI, diabetes mellitus status, SBP, and antihypertension medication usage (Supplementary Table S1). However, the between-group difference of reduction in DBP was insignificant at week 8 in patients who were male or with BMI ≥25 kg/m^2^ at baseline. Compared to control group, HI therapy did not provide beneficial effect on mean DBP levels at week 8 and week 16 for participants aged <65 years, with diabetes mellitus history, or taking >1 antihypertensives. Notably, there was no significant between-group difference of reduction in DBP during 24 weeks follow-up period for subjects with baseline SBP <140 mm Hg. The results of settling missing follow-up data by multiple-imputation method did not differ from the main analysis (Supplementary Table S2). The findings remained mostly consistent after re-applying analysis in all subjects with the absence of PSM (Supplementary Table S3). Similarly, using less restrictive criteria of patient selection led to inappreciable changes (Supplementary Table S4).

### Safety evaluation

Generally, 47 (4.0%) patients in HI group experienced 68 AEs and 61 (5.2%) patients in control group experienced 79 AEs with no significant difference (*p* = 0.168). The most common AE in both groups was dizziness with 15 events occurring in 15 (1.3%) participants in HI group and 25 events occurring in 24 (2.0%) participants in control group. The incidences of all AEs (dizziness, headache, palpitations, asthenia, edema, flushing, cough, etc.) were similar between two groups with all *p*-value >0.05. More details of AE are shown at Table 2.

**Table 2:** Comparison of the adverse events between the two groups.

Variables		HI group (*N* = 1,182)	Control group (*N* = 1,182)	*P*-value
Dizziness	No. of events	15	25	0.146
	No. (%)	15 (1.3)	24 (2.0)	
Headache	No. of events	8	11	0.490
	No. (%)	8 (0.7)	11 (0.9)	
Palpitations	No. of events	6	5	0.526
	No. (%)	6 (0.5)	4 (0.3)	
Asthenia	No. of events	7	7	0.563
	No. (%)	5 (0.4)	7 (0.6)	
Edema	No. of events	7	9	0.616
	No. (%)	7 (0.6)	9 (0.8)	
Flushing	No. of events	4	4	1.000
	No. (%)	4 (0.3)	4 (0.3)	
Cough	No. of events	6	6	0.762
	No. (%)	6 (0.5)	5 (0.4)	
Dyspnea	No. of events	2	4	0.687
	No. (%)	2 (0.2)	4 (0.3)	
Dyspepsia	No. of events	4	2	1.000
	No. (%)	2 (0.2)	2 (0.2)	
Others	No. of events	9	6	0.404
	No. (%)	8 (0.7)	5 (0.4)	

Note: All data are presented as *n* (%).

Abbreviation: HI, hydrogen inhalation.

## Discussion

This multicenter, observational, retrospective, real world study aims at examining the effectiveness and safety of HI as an additional therapy in Chinese hypertensive patients. We found that the HI is related to greater improvement in mean BP and amelioration of BP control rate with comparable incidence of AE during 24 weeks compared to routine antihypertensive medication.

Recently, a clinical trial reported that compared to placebo group, the intervention of a low-dose 66% H2/33% O2 mixture in 60 patients with hypertension for 2 weeks significantly reduced right arm SBP by 4.8 mm Hg and improved nighttime DBP by 2.7 mm Hg, while other BP remained no marked change (25). In current study, both the mean SBP and DBP levels remain decreasing and the BP control rates keep increasing for HI group and control group during 24-week follow-up period. Meanwhile, patients treated with HI therapy receive more favorable effects in BP amelioration and attainment of controlled BP compared to participants treated with routine hypertensive medication for each visit point. The results of subgroup and sensitivity analysis are mostly the same as the main analysis, which confirms the robustness of our findings. Despite the primary outcome of our study is consistent with previous RCT, the different population of patients, follow-up duration, and intervention strategy of molecular hydrogen may account for the distinct results.

The most common AE is dizziness both in HI group and control group, and the incidences of all AEs are similar between two groups during follow-up period, which indicts that the HI therapy has high safety profile. Our findings are consistent with many previous RCTs, which reported no AE during HI intervention (16, 28, 29, 35). Lacking of cytotoxicity even at high concentration, hydrogen played as a role in the gas mixture for prevention of decompression sickness in extremely deep diving (36–39). 98.87% H2 and 1.13% O2 at 19.1 atm also showed no toxic effect (40). Meanwhile, as our intestinal bacteria ferment indigestible carbohydrates, our blood and breath naturally contain hydrogen gas (41). Thus, patients in HI group could apply hydrogen therapy freely without strict limitation of frequency or duration.

It is hard to directly assess the compliance of patients in this retrospective study. Nevertheless, the time period of HI per week has an increasing trend for participants receiving hydrogen therapy during 24-week follow-up period, which might reflect the favorable compliance of patients for HI treatment.

Although HI therapy has been proved to be an effective BP lowering strategy, the underlaying mechanism remains vague. First, the magnitude of antioxidant intervention in hypertension management has been highlighted in several clinical studies (42). Acting as an essential antioxidant enzyme to adjust reactive oxygen species (ROS) levels, superoxide dismutase (SOD) has lower activity for participants with hypertension (43). Therefore, oxidative stress appears to be generally existing in hypertension. Ohsawa et al. (44) had reported that the hydrogen presented therapeutic antioxidant effect by selectively eliminating ROS. The oxidative stress of several animal models with high BP was alleviated by hydrogen therapy (22, 45). Therefore, molecular hydrogen may ameliorate hypertension via reducing oxidative stress. Second, The renin–angiotensin–aldosterone system (RAAS) consists of several relevant hormones which function together to modulate BP (46). The angiotensin II, formed by the interaction of renin and angiotensin converting enzyme, leads to vasoconstriction, and further triggers elevation of BP. Meanwhile, it induces aldosterone secretion, which raises body fluid volume and BP level by promoting kidney to reabsorb sodium and water (47, 48). In addition, Whitworth et al. (49) reported that the incidence of hypertension and cardiovascular events was related to increased stress hormone cortisol. Previous clinical study found that HI therapy was associated with significant reduction in the plasma angiotensin II, aldosterone, aldosterone-to-renin ratio, and cortisol levels, which at least partly accounts for antihypertensive effect of molecular hydrogen (25). Third, inflammation acts as an essential part in hypertensive pathophysiology. It was reported that several new targets were developed for hypertension, such as specific immune cell types, toll-like receptors, and cytokines (50). The inflammatory status and BP level were improved by using hydrogen-rich hemodialysis solutions for 21 hemodialysis patients (51). The pro-inflammatory cytokines level was ameliorated by hydrogen-rich saline in rat with pulmonary hypertension (52). These findings indict that molecular hydrogen alleviates inflammatory reaction in hypertension. Thus, the therapeutic effect of HI for hypertension might be due to its anti-inflammatory feature.

In current clinical trials and real-life application, the most frequent methods of intaking molecular hydrogen are drinking HRW and hydrogen gas inhalation. However, it was reported that 6-month intervention of HRW or HI had an different influence upon 13 serum biochemical parameters in rats (53). In addition, different concentrations of hydrogen gas resulted in distinct therapeutic effects in rats with ischemia–reperfusion injury (54). Thus, further study is demanded to assess the optimal concentration and method for molecular hydrogen therapy.

Hypertension usually require long-term medication intervention continuously. A systematic review indicated that the BP of three quarters of patients who withdrew antihypertensive treatment would return to the level requiring reuse of therapy (55). Although HI therapy demonstrates therapeutic effect for hypertension in this study, its persistence after discontinuation still demands further assessment in future researches.

This study contains several limitations: First, the unmeasured confounding and sample bias are inevitable in this retrospective real-world study even after the application of PSM, due to the lack of randomization. Second, the incidence of AE may be underestimated in this study, as the evaluation is based on the medical records. Third, this study solely selects Chinese subjects with hypertension, and the results could hardly be fully generalizable to populations in other countries. Fourth, several essential factors related to treatment are unmeasured in current research, such as the blood concentration of H2 and O2, and the persistence status of antihypertensive effect after HI discontinuation. Finally, the optimal strategy and dosage of hydrogen intervention for hypertension require deeper investigation. Nevertheless, we evaluated the beneficial effects and safety of HI therapy in hypertensive patients in greater follow-up period and larger sample size, enriching the evidence base of hydrogen medicine in hypertension.

In conclusion, our study indicts that the 24-week HI therapy significantly ameliorates BP level and increases the BP control rate with similar incidence of AE compared with control group in Chinese hypertensive patients. It demonstrates an effective and possible antihypertensive therapy for current clinical practice.

## Data Availability

The raw data supporting the conclusions of this article will be made available by the authors, without undue reservation.
