# Supplementary material for: Effectiveness and safety of hydrogen inhalation therapy as an additional treatment for hypertension in real-world practice: a retrospective, observational study in China
Source: Front Cardiovasc Med. 2024 Nov 12;11:1391282. doi: 10.3389/fcvm.2024.1391282 (PMC11588699; doi:10.3389/fcvm.2024.1391282)
Supplement: Supplementary file 1 [file Table1.docx]

**Supplementary materials**

Table S1 Subgroup analyses for change in blood pressure during the study according to participant characteristics at baseline

| **Parameters** | **The mean between-group difference for blood pressure** | | |
| --- | --- | --- | --- |
|  | **Week 8** | **Week 16** | **Week 24** |
| **Change in mean systolic blood pressure from baseline, mm Hg** | | | |
| Baseline age, years | | | |
| <65 | -4.33 (-6.50 to -2.15) | -5.43 (-7.51 to -3.35) | -7.15 (-9.16 to -5.14) |
| ≥65 | -5.20 (-8.28 to -2.11) | -8.05 (-11.24 to -4.86) | -8.60 (-11.63 to -5.57) |
| Sex | | | |
| Male | -4.69 (-7.36 to -2.03) | -7.04 (-9.60 to -4.47) | -8.54 (-11.02 to -6.06) |
| Female | -4.62 (-7.29 to -1.95) | -6.41 (-9.07 to -3.74) | -7.19 (-9.69 to -4.69) |
| Baseline BMI, kg/m2 | | | |
| <25 | -5.27 (-8.36 to -2.18) | -7.78 (-10.96 to -4.59) | -8.50 (-11.56 to -5.45) |
| ≥25 | -4.63 (-6.82 to -2.44) | -5.96 (-8.04 to -3.88) | -7.55 (-9.54 to -5.55) |
| Diabetes | | | |
| Yes | -4.12 (-6.76 to -1.49) | -5.36 (-7.89 to -2.82) | -7.37 (-9.78 to -4.96) |
| No | -5.40 (-7.55 to -3.25) | -7.50 (-9.61 to -5.39) | -8.31 (-10.36 to -6.27) |
| Baseline SBP, mmHg | | | |
| <140 | -2.93 (-5.29 to -0.57) | -4.29 (-6.53 to -2.06) | -7.33 (-9.31 to -5.35) |
| ≥140 | -5.63 (-7.54 to -3.72) | -8.10 (-9.95 to -6.26) | -8.13 (-9.85 to -6.41) |
| Use of antihypertensive drug | | | |
| ≤1 | -5.54 (-8.42 to -2.66) | -9.20 (-12.11 to -6.30) | -8.46 (-11.25 to -5.66) |
| >1 | -4.53 (-6.75 to -2.32) | -4.93 (-7.07 to -2.79) | -7.65 (-9.69 to -5.62) |
| **Change in mean diastolic blood pressure from baseline, mm Hg** | | | |
| Baseline age, years | | | |
| <65 | -1.04 (-2.68 to 0.59) | -1.39 (-3.01 to 0.22) | -1.70 (-3.25 to -0.14) |
| ≥65 | -3.01 (-5.26 to -0.77) | -4.05 (-6.47 to -1.64) | -4.81 (-7.15 to -2.46) |
| Sex | | | |
| Male | -1.70 (-3.66 to 0.25) | -2.59 (-4.57 to -0.60) | -2.72 (-4.63 to -0.80) |
| Female | -2.00 (-3.95 to -0.04) | -2.59 (-4.57 to -0.61) | -3.15 (-5.05 to -1.24) |
| Baseline BMI, kg/m2 | | | |
| <25 | -2.88 (-5.12 to -0.64) | -3.59 (-6.00 to -1.18) | -4.44 (-6.80 to -2.08) |
| ≥25 | -1.38 (-3.02 to 0.26) | -1.90 (-3.51 to -0.29) | -2.14 (-3.68 to -0.59) |
| Diabetes | | | |
| Yes | -1.30 (-3.34 to 0.75) | -1.45 (-3.48 to 0.58) | -2.03 (-3.99 to -0.07) |
| No | -2.43 (-4.03 to -0.83) | -3.23 (-4.87 to -1.58) | -3.56 (-5.15 to -1.97) |
| Baseline SBP, mmHg | | | |
| <140 | 0.37 (-1.72 to 2.47) | -0.25 (-2.35 to 1.84) | -1.58 (-3.59 to 0.44) |
| ≥140 | -2.97 (-4.59 to -1.35) | -3.97 (-5.62 to -2.32) | -3.46 (-5.08 to -1.84) |
| Use of antihypertensive drug | | | |
| ≤1 | -2.70 (-4.79 to -0.62) | -4.31 (-6.53 to -2.09) | -3.77 (-5.92 to -1.62) |
| >1 | -1.55 (-3.22 to 0.13) | -1.35 (-2.99 to 0.30) | -2.51 (-4.09 to -0.94) |

Note: All data are presented as mean (95% CI).

Abbreviation: BMI, body mass index; SBP, systolic blood pressure.

Table S2 Effectiveness of HI on blood pressure levels: sensitivity analysis of imputing missing data during the study

| **Parameters** | **HI group** | **Control group** | **Difference/OR*** |
| --- | --- | --- | --- |
| **Change in mean systolic blood pressure from baseline, mm Hg** | | | |
| Week 8 | -5.87 (-7.13 to -4.60) | -1.53 (-2.79 to -0.26) | -4.34 (-6.12 to -2.56) |
| Week 16 | -8.71 (-9.91 to -7.51) | -3.13 (-4.34 to -1.91) | -5.58 (-7.28 to -3.88) |
| Week 24 | -11.30 (-12.43 to -10.17) | -4.01 (-5.15 to -2.88) | -7.28 (-8.86 to -5.71) |
| **Change in mean diastolic blood pressure from baseline, mm Hg** | | | |
| Week 8 | -3.20 (-4.14 to -2.25) | -1.43 (-2.40 to -0.46) | -1.76 (-3.10 to -0.43) |
| Week 16 | -4.46 (-5.37 to -3.56) | -1.83 (-2.76 to -0.90) | -2.63 (-3.92 to -1.35) |
| Week 24 | -6.28 (-7.13 to -5.44) | -3.31 (-4.20 to -2.42) | -2.98 (-4.20 to -1.76) |
| **Percentage of participants with controlled blood pressure ǂ** | | | |
| Week 8 | 38.1 (35.3 to 40.8) | 30.2 (27.6 to 32.8) | 1.42 (1.20 to 1.69) |
| Week 16 | 51.4 (48.6 to 54.3) | 35.6 (32.9 to 38.4) | 1.92 (1.62 to 2.26) |
| Week 24 | 60.8 (58.0 to 63.6) | 40.1 (37.3 to 42.9) | 2.32 (1.97 to 2.74) |

Note: All data are presented as mean (95% CI) or percentage (95% CI). * Difference (HI group- control group) is shown for mean blood pressure changes from baseline, and OR (HI group: control group) is shown for percentages of blood pressure control rate. ǂ Controlled blood pressure was considered as systolic blood pressure < 140 mmHg and diastolic blood pressure < 90 mmHg.

Abbreviation: HI, hydrogen inhalation; OR, odds ratio.

Table S3 Effectiveness of HI on blood pressure levels in the overall study participants without propensity score matching

| **Parameters** | **HI group** | **Control group** | **Difference/OR*** |
| --- | --- | --- | --- |
| **Change in mean systolic blood pressure from baseline, mm Hg** | | | |
| Week 8 | -5.74 (-6.80 to -4.67) | -2.12 (-3.04 to -1.20) | -3.62 (-5.03 to -2.21) |
| Week 16 | -8.34 (-9.39 to -7.29) | -3.31 (-4.20 to -2.43) | -5.03 (-6.40 to -3.65) |
| Week 24 | -10.87 (-11.90 to -9.85) | -4.32 (-5.20 to -3.44) | -6.55 (-7.91 to -5.20) |
| **Change in mean diastolic blood pressure from baseline, mm Hg** | | | |
| Week 8 | -2.92 (-3.74 to -2.11) | -0.46 (-1.15 to 0.23) | -2.46 (-3.53 to -1.40) |
| Week 16 | -3.93 (-4.70 to -3.16) | -0.86 (-1.55 to -0.17) | -3.07 (-4.10 to -2.04) |
| Week 24 | -5.96 (-6.74 to -5.18) | -2.82 (-3.49 to -2.15) | -3.14 (-4.17 to -2.10) |
| **Percentage of participants with controlled blood pressure ǂ** | | | |
| Week 8 | 37.3 (35.0 to 39.7) | 22.7 (21.0 to 24.4) | 2.03 (1.76 to 2.33) |
| Week 16 | 52.4 (49.9 to 54.9) | 26.9 (25.0 to 28.7) | 3.00 (2.61 to 3.44) |
| Week 24 | 59.5 (57.0 to 62.1) | 35.2 (33.1 to 37.2) | 2.71 (2.36 to 3.12) |

Note: All data are presented as mean (95% CI) or percentage (95% CI). * Difference (HI group- control group) is shown for mean blood pressure changes from baseline, and OR (HI group: control group) is shown for percentages of blood pressure control rate. ǂ Controlled blood pressure was considered as systolic blood pressure < 140 mmHg and diastolic blood pressure < 90 mmHg.

Abbreviation: HI, hydrogen inhalation; OR, odds ratio.

Table S4 Effectiveness of HI on blood pressure levels: sensitivity analysis of applying patient selection criteria with less restriction

| **Parameters** | **HI group** | **Control group** | **Difference/OR*** |
| --- | --- | --- | --- |
| **Change in mean systolic blood pressure from baseline, mm Hg** | | | |
| Week 8 | -5.33 (-6.39 to -4.26) | -0.69 (-1.79 to 0.41) | -4.63 (-6.16 to -3.10) |
| Week 16 | -8.45 (-9.49 to -7.40) | -2.69 (-3.75 to -1.64) | -5.75 (-7.24 to -4.27) |
| Week 24 | -10.39 (-11.38 to -9.39) | -3.54 (-4.57 to -2.52) | -6.84 (-8.27 to -5.42) |
| **Change in mean diastolic blood pressure from baseline, mm Hg** | | | |
| Week 8 | -2.34 (-3.13 to -1.55) | -0.46 (-1.23 to 0.31) | -1.88 (-2.98 to -0.77) |
| Week 16 | -3.93 (-4.70 to -3.16) | -1.16 (-1.93 to -0.40) | -2.77 (-3.86 to -1.68) |
| Week 24 | -5.99 (-6.74 to -5.25) | -2.73 (-3.49 to -1.97) | -3.26 (-4.33 to -2.20) |
| **Percentage of participants with controlled blood pressure ǂ** | | | |
| Week 8 | 40.5 (38.2 to 42.9) | 29.8 (27.6 to 32.0) | 1.60 (1.39 to 1.85) |
| Week 16 | 51.7 (49.2 to 54.2) | 36.9 (34.5 to 39.2) | 1.84 (1.59 to 2.11) |
| Week 24 | 57.9 (55.4 to 60.5) | 40.8 (38.4 to 43.3) | 2.00 (1.73 to 2.31) |

Note: All data are presented as mean (95% CI) or percentage (95% CI). * Difference (HI group- control group) is shown for mean blood pressure changes from baseline, and OR (HI group: control group) is shown for percentages of blood pressure control rate. ǂ Controlled blood pressure was considered as systolic blood pressure < 140 mmHg and diastolic blood pressure < 90 mmHg.

Abbreviation: HI, hydrogen inhalation; OR, odds ratio.
